# Plastid Phylogenomic Analysis of Tordylieae Tribe (Apiaceae, Apioideae)

**DOI:** 10.3390/plants11050709

**Published:** 2022-03-07

**Authors:** Tahir Samigullin, Maria Logacheva, Elena Terentieva, Galina Degtjareva, Michael Pimenov, Carmen Valiejo-Roman

**Affiliations:** 1 Department of Evolutionary Biochemistry, A. N. Belozersky Research Institute of Physicochemical Biology, Lomonosov Moscow State University, Leninskie Gory 1–40, 119992 Moscow, Russia; maria.log@gmail.com (M.L.); vallejo@belozersky.msu.ru (C.V.-R.); 2 Center of Life Sciences, Skolkovo Institute of Science and Technology, Bolshoy Boulevard 30, Bld. 1, 121205 Moscow, Russia; 3 Botanical Garden, Faculty of Biology, Lomonosov Moscow State University, Leninskie Gory 1/12, 119992 Moscow, Russia; el.terenteva@mail.ru (E.T.); degavi@mail.ru (G.D.); mgpimenov@mail.ru (M.P.)

**Keywords:** plastome evolution, inverted repeats, contraction, expansion, Apiaceae, Tordylieae, phylogenomics

## Abstract

Based on the nrDNA ITS sequence data, the Tordylieae tribe is recognized as monophyletic with three major lineages: the subtribe Tordyliinae, the *Cymbocarpum* clade, and the *Lefebvrea* clade. Recent phylogenomic investigations showed incongruence between the nuclear and plastid genome evolution in the tribe. To assess phylogenetic relations and structure evolution of plastomes in Tordylieae, we generated eleven complete plastome sequences using the genome skimming approach and compared them with the available data from this tribe and close relatives. Newly assembled plastomes had lengths ranging from 141,148 to 150,103 base pairs and contained 122–127 genes, including 79–82 protein-coding genes, 35–37 tRNAs, and 8 rRNAs. We observed substantial differences in the inverted repeat length and gene content, accompanied by a complex picture of multiple J_LA_ and J_LB_ shifts. In concatenated phylogenetic analyses, Tordylieae plastomes formed at least three not closely related lineages with plastomes of the *Lefebvrea* clade as a sister group to plastomes from the Selineae tribe. The newly obtained data have increased our knowledge on the range of plastome variability in Apiaceae.

## 1. Introduction

During the most recent decade, active studies of angiosperm plastid genomes (plastomes) have shown that plastomes are relatively conserved but dynamic in terms of gene content and structural features, and many amazing examples of radical enlargement or reduction and rearrangements have been uncovered [1,2,3,4]. Most of the flowering plants, however, possess plastomes evolving in a calm manner and exhibit structural diversity only in the position of inverted repeats (IR) boundaries. In addition to the “ebb and flow” of the inverted repeats borders [5], the incorporation of “foreign” DNA of mitochondrial or uncertain origin is being reported more and more often [2,6].

In Apiaceae plastomes, gene order and genome structure are significantly conserved [7,8,9,10,11,12,13,14,15,16,17,18,19,20,21,22,23,24,25,26,27,28]. However, while for land plant’s plastomes (from early non-vascular plants to angiosperms), a general trend of IR enlarging can be seen [1,29], in the Apioideae subfamily of Apiaceae, a contrary tendency of IR shrinking is observed [26,30]. The shortest inverted repeats are concentrated within a distally branching group of clades (apioid superclade), uniting several tribes and clades with unclear relationships, including the Tordylieae tribe [31,32].

Tordylieae was established as a tribe, consisting of the genera *Tordylium* L., *Condylocarpus* Hoffm., *Krubera* Hoffm., and *Hasselquistia* L., by Koch (1824) [33]. Circumscription of the tribe was not stable and changed significantly in rank, name, and genera content, and in the system proposed by Pimenov and Leonov (1993) [34], Tordylieae included 23 genera. In addition to several large genera (*Heracleum* L., *Pastinaca* L., *Tetrataenium* (DC.) Manden., *Tordylium*, *Semenovia* Regel & Herder, the tribe also includes mono and oligotypic genera (*Mandenovia* Alava, *Vanasushava* P.K.Mukh. & Constance, *Symphyoloma* C.A.Mey., *Pastinacopsis* Golosk., *Tordyliopsis* DC., *Ducrosia* Boiss., *Kalakia* Alava, *Tricholaser* Gilli, etc.), almost all of them are rare plants, known from limited locations. Although Tordylieae, as a whole, was not the subject of special molecular phylogenetic studies, a lot of information regarding relationships and generic circumscription in the tribe has been obtained [35,36,37,38,39,40,41,42,43,44], and this information was summed up in the nrITS-based classification of the subfamily Apioideae [31]. According to this classification and the data obtained later [45,46,47], the tribe is recognized as monophyletic with the following major lineages: subtribe Tordyliinae Engl., containing such genera as *Heracleum*, *Malabaila* Hoffm., *Pastinaca*, *Semenovia*, and *Tordylium*; the *Cymbocarpum* clade, comprising the genera *Cymbocarpum* DC., *Kalakia*, and *Ducrosia*; and the *Lefebvrea* clade consisting of the “African peucedanoid group”. However, Tordylieae appeared to not be monophyletic in a recent broad nuclear phylogenomic coalescent analysis of sequences obtained using the universal Angiosperm353 probe set: the *Cymbocarpum* clade separated from the rest of Tordylieae, though quartet scores indicated notable discordance in the gene trees and local posterior probabilities supporting the position of the *Cymbocarpum* clade were not high [48].

Individual plastid markers (intron sequences of *rps16*, *rpl16*, *rpoC1* genes) often did not show sufficient variability to provide resolution both within the Tordylieae and between the adjacent tribes or produced trees incongruent with the nrITS phylogeny [35,49,50]. A recent plastid phylogenomic investigation claimed new robust evidence for incongruence between the nuclear genome and plastome evolution in Tordylieae [20,26]. Within the Tordyliinae subtribe, *Pastinaca* and *Heracleum* formed a well-supported subclade (designated as Tordyliinae I) as a sister group to the Selineae tribe. Another subclade (Tordyliinae II) included *Semenovia* and *Tetrataenium* and was resolved as a part of a larger clade, which was a sister group to the *Sinodielsia* clade. These studies did not include representatives of the *Lefebvrea* and *Cymbocarpum* clades but revealed a noticeable difference in the plastome structure of Tordyliinae I and Tordyliinae II members. They experienced a similar IR expansion at the LSC/IRa border (J_LA_ shift), but the insertion of the *trnH* and *psbA* sequences occurred in different spacers: the *ycf2*-*trnL*(CAA) spacer in Tordyliinae I and *trnV*(GAC)-*rrn16* spacer in Tordyliinae II. Similar rearrangements caused by the J_LA_ shift were also mentioned in several non-monocot angiosperm families (Actinidiaceae [51], Chenopodiaceae [52], Linaceae [53], Acanthaceae [54]) and in other Apiaceae species [26,55]. Moreover, two of ten plastomes of *Angelica sinensis* (Oliv.) Diels (member of *Sinodielsia* clade from the apioid superclade), and two of three available *Semenovia* plastomes showed absence of the insertion [21,28]. Therefore, its appearance, distribution across plastomes from members of the Tordylieae tribe and allied taxa that were yet unsampled, and utility of the specific plastome rearrangements as phylogenetic markers are of special interest. In this work, in order to address questions of phylogenetic relationships and structure evolution of plastomes in Tordylieae, and utility of specific rearrangements in the plastid genome as phylogenetic markers, we determined eleven complete plastome sequences (from *Dasispermum suffruticosum* (P.J.Bergius) B.L.Burtt, *Ducrosia anethifolia* Boiss., *Kalakia marginata* (Boiss.) Alava, *Mandenovia komarovii* (Manden.) Alava, *Notobubon galbanum* (L.) Magee, *Pastinaca pimpinellifolia* M.Bieb., *Symphyoloma graveolens* C.A.Mey., *Tordylium lanatum* Boiss., *Tordylium maximum* L., *Tordylium pestalozzae* Boiss., and *Zosima korovinii* Pimenov) using a genome skimming approach and compared them with the available data from the tribe and close relatives. Phylogenomic analyses and tracking of specific rearrangements showed polyphyly of Tordylieae plastomes accompanied with a complex picture of multiple J_LA_ and J_LB_ shifts.

## 2. Results and Discussion

### 2.1. General Overview of Plastomes

All newly assembled plastomes of species from Tordylieae possessed a quadripartite structure typical for angiosperms [56] with an overall length ranging from 141,148 (*D. anethifolia*) to 150,103 (*T. maximum*) base pairs (bp) (Table 1). The LSC regions ranged from 99,620 (*Z. korovinii*) to 91,637 bp (*T. maximum*) in size, whereas the SSC ranged from 17,676 (*T. maximum*) to 16,931 bp (*D. suffruticosum*); the pair of inverted repeats separated by the small single-copy region ranged from 20,395 (*T. maximum*) to 12,493 bp (*T. pestalozzae*) (Table 1). The overall GC content varied slightly between 37.1% and 37.5%. The eleven plastomes contained 122–127 genes, including 79–82 protein-coding genes, 35–37 tRNA genes, and 8 rRNA genes (Supplementary Table S1). All but *T. pestalozzae* plastid genomes contained two pseudogenes, in all plastomes, one was a truncated copy of the *ycf1* gene at the SSC/IRb junction (J_SB_), while another was either a part of the *ycf2* gene at the LSC/IRa junction (J_LA_) (*D. suffruticosum*, *N. galbanum*, and *T. maximum*), a fragment of the *psbA* gene at the LSC/IRb junction (J_LB_) (*M. komarovii*, *S. graveolens*, *P. pimpinellifolia*, *Z. korovinii*, *K. marginata*, and *D. anethifolia*), or an exon of the *ndhB* gene at J_LA_ (*T. lanatum*). The *Tordylium pestalozzae* plastome contained only a truncated copy of the *ycf1* gene.

The plastome of *Ducrosia* showed a 489-bp inversion in the LSC region; it affects the orientation of the three tRNAs (*trnE*, *trnY*, *trnD*). This inversion was flanked by 39 complementary bases, and similar inversions in the same region were also revealed in other Apioideae plastomes not closely related to each other: *Cyclospermum leptophyllum* (Urb.) Constance (Pyramidoptereae tribe), *Carum carvi* L. (Careae tribe) [55], *Angelica gigas* Nakai, and *Angelica morii* Hayata (Selineae tribe) [25]. Convergent inversions are not rare in plastid genomes and have also been documented in other families, e.g., Ranunculaceae [57], Oleaceae [58], Asteraceae [59], Fabaceae [60,61,62]. Moreover, in a recent study, Charboneau et al. noticed possible heteroplasmy in *Astragalus* for the presence of the inversions [62], so actually, the inversion of the *trnE*-*trnY*-*trnD* region in *Ducrosia* and other Apioideae plastomes might be present in both orientations, with one variant being in a minor proportion.

### 2.2. Inverted Repeat Contractions and Expansions

**Figure 1:**
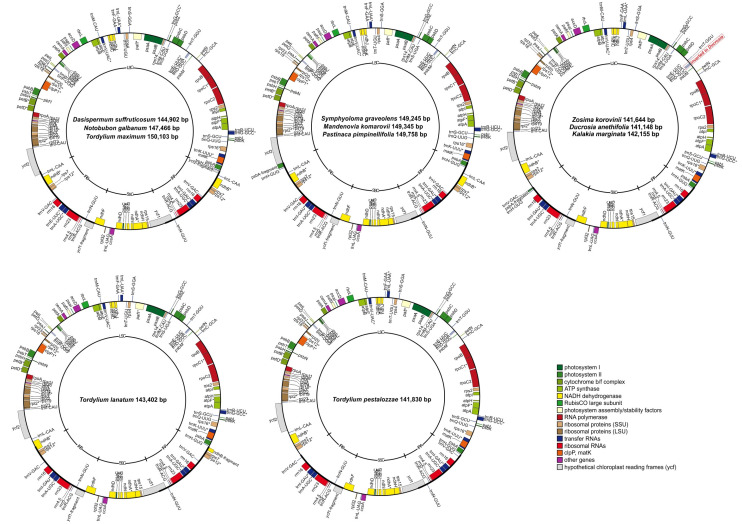
Circular gene maps of eleven plastid genomes of Tordylieae tribe representatives. Genes plotted outside the circle are transcribed counterclockwise, inside genes—clockwise. Genes are colored according to their function, intron-containing genes are marked with asterisks. LSC = large single-copy region, SSC = small single-copy region, IRA and IRB = inverted repeats A and B, respectively.

Numerous indels have been accumulated in the studied plastomes, but their contribution to the genome length diversity is not high. This can be seen from the size variation of the SSC region (from 16,931 to 17,676 bp), the borders of which may be considered relatively stable, as they are in the same relative gene position. Substantial differences in length across the eleven plastomes and differences in total gene number (122, 124, 126, 127) are caused by contraction and expansion of the inverted repeats, reflecting their border shifts (Figure 1).

The longest IRs belong to *T. maximum* with J_LB_ in *ycf2*; J_LB_ in *D. suffruticosum*; and *N. galbanum* is situated within the same gene. In the plastomes of *M. komarovii*, *S. graveolens*, and *P. pimpinellifolia*, the inverted repeats are shortened, and J_LB_ is followed by the *ycf2* gene sequence. The shortest IRs are found in the *Z. korovinii* plastome, the position of J_LB_ is several base pairs after the *trnV*(GAC) gene, and it is shared with *D. anethifolia* and *K. marginata* plastomes. In the plastome of *T. pestalozzae*, the inverted repeat includes the *trnV*(GAC) gene, while in the *T. lanatum* plastome the inverted repeat is expanded further with J_LB_ situated within the intron of the *ndhB* gene. The last two J_LB_ shifts are observed for the first time in the complete plastid genome studies and represent new examples of how variable the plastome organization within Apiaceae can be. Earlier studies using restriction fragment length polymorphism analysis allowed the definition of nine J_LB_ junction types [30], while complete plastome sequences recovered new types [9,10,20,24,25,26], and nowadays, their number exceeds fifteen [26].

The IRa/LSC border is generally less prone to shift towards LSC because of the limited space where the insertion of the LSC sequence at J_LB_ is not fatal. Indeed, rare known examples of such J_LA_ shift from non-monocot angiosperms demonstrate the appearance of *trnH*(GUG) (sometimes with a partial sequence of *psbA* or a longer sequence, including *matK*, *rps16*, and *trnQ*(UUG) genes) in the *rpl23*-*trnI* spacer (in Actinidiaceae [51], Chenopodiaceae [52]), in the *trnI*-*ycf2* spacer (in Linaceae [53]), in the *ycf2*-*trnL* spacer (in Acanthaceae [54]), and even invasion of the *trnH* sequence into the *S10* operon was possible in *Elaeagnus* L. species [63], several *Aristolochia* L. species [64], *Drimys granadensis* L.f. [65], *Stylidium debile* F.Muell. [66], and *Poeppiga procera* (Poepp. ex Spreng.) C.Presl [67]. In our study, we have detected J_LA_ shifts resulting in the inclusion of *trnH* and a partial sequence of *psbA* in IR with their appearance at J_LB_ in the *ycf2*-*trnL* spacer in *S. graveolens*, *M. komarovii*, and *P. pimpinellifolia* plastomes similar to what was recently observed in two *Heracleum* and *Pastinaca sativa* L. plastomes [19,26]. Similar J_LA_ shifts resulted in the appearance of the *trnH* and *psbA* partial sequence at J_LB_ in the *trnV*-*rrn16* spacer in *Z. korovinii*, *D. anethifolia*, and *K. marginata* plastomes, as it was already observed in *Semenovia* and *Tetrataenium* [20]. During annotation of plastomes of *T. lanatum* and *T. pestalozzae*, we did not find *psbA* or *trnH* gene sequences in the *trnV*-*rrn16* spacer; however, the spacer contained an insertion of the non-coding sequence (957 and 1153 bp, respectively) that has significant similarity with the part of the *rrn16*-*trnH* spacer of the plastomes of *Zosima*, *Ducrosia*, and *Kalakia*. The insertion in the *trnV*-*rrn16* spacer was verified and confirmed using PCR-amplification and Sanger sequencing in *Z. korovinii*, *D. anethifolia*, *K. marginata*, *T. lanatum*, and *T. pestalozzae* plastomes.

In the analyzed plastomes with insertions, which contain a partial *psbA* sequence, the LSC/IRb borders are notably stuck to the beginning of the insertions because the IRb expansion into LSC would inevitably cause the *psbA* gene disruption. At this point, the genomes of *T. pestalozzae* and *T. lanatum* demonstrate a departure of the J_LB_ junction from the insertion place and hide an actual trajectory of the IR borders locomotion: they experienced expansion by the J_LA_ shift followed by the acquisition of the insertion in the *trnV*-*rrn16* spacer, then, two expansions of the J_LB_ shifts—either separately in *T. pestalozzae* and *T. lanatum*, or one common for both and further expansion in *T. lanatum* only. Such “hidden” insertions are seen in alignment, but they may be easily overlooked when J_LA_ shifts are screened by the annotated genes in the plastomes of other plants. For example, several Apiaceae plastomes in the Selineae tribe contain an insertion (ranging in length from 794 bp in *Saposhnikovia divaricata* (Turcz.) Schischk. to 1010 bp in *Kitagawia praeruptora* (Dunn) Pimenov [=*Peucedanum praeruptorum* Dunn]) at the 3′-end of the *ycf2* gene, which is highly variable across land plants. This insertion retains similarity with the insertions in the *trnV*-*rrn16* and *ycf2*-*trnL* spacers (Supplement Figure S1) of about 180 bp at its 3′-end. It might also have been generated by the J_LA_ shift and awaits further detailed analysis.

### 2.3. Phylogenetic Analysis of Tordylieae Plastomes

Phylogenetic relationships were inferred using two matrices: complete plastome sequences with one inverted repeat excluded (“long” matrix) and protein-coding sequences (“CDS” matrix). The long data matrix contained only reliably aligned positions from intergenic regions, genes, and introns with the presence of gaps in less than half of the sequences. The only completely excluded intergenic spacer was the pseudo-*ycf1*-*ndhF* spacer because of high variability. Special attention was paid to the presence of small inversions as they increase the level of homoplasy and may affect the inferred topology, model parameters, and branch length estimation, as well as branch support assessment even in the full plastome data analyses [68,69,70]. Small inversions are widespread in the plastomes across the land plants [68,69,71] and have also been reported in Apioideae [15,42]. In our dataset, nineteen small inversions with a length ranging from 3 to 62 bases were identified, and one of them was found within the *ycf1* gene. To restore the homology of the nucleotide bases, these inversions were reverse complemented. The resulting data matrix had 127,509 characters and contained 15,610 variable positions, 5326 of which were parsimony informative. The CDS data matrix contained only protein-coding sequences extracted from the long matrix; it had 67,875 characters and contained 6414 variable positions, 2144 of which were parsimony informative.

**Figure 2:**
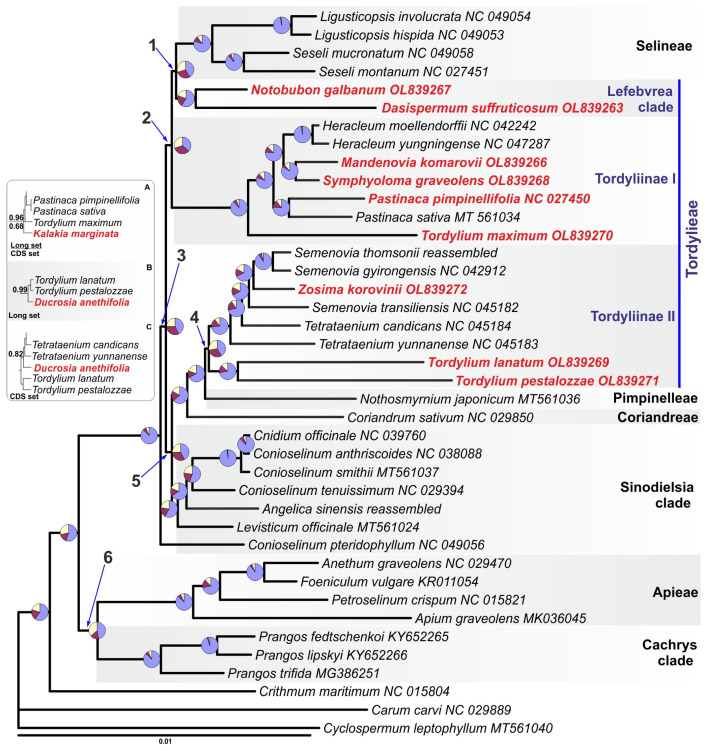
Maximum *a posteriori* probability tree (PP = 1) inferred with Bayesian analysis of complete plastome sequences from 40 representatives of the apioid superclade. Plastomes derived for this study are marked with red. Support for enumerated branches 1–6 is presented in Table 2; others gained the highest support in all analyses. The pie chart represents the site concordance (purple)/discordance (red and yellow) factor. The inset shows *Kalakia* (**A**) and *Ducrosia* (**B**,**C**) placement and support when included in Bayesian analyses of CDS (**A**,**C**) and long (**A**,**B**) data sets.

Our preliminary phylogenetic analyses showed that *Ducrosia* and *Kalakia* behaved as rogue-taxa [72] and destabilized the overall tree topology because of the absence of closest relatives, as we suppose. In the absence of *Ducrosia* and *Kalakia*, both unpartitioned and partitioned phylogenetic analyses resulted in the same topology; therefore, we present a phylogenetic tree reconstructed without *Ducrosia* and *Kalakia* (Figure 2) with insets indicating positions that they occupy when included in Bayesian analyses.

While most of internal branches of the tree gained the highest support irrespective of the used partitioning scheme and data set in all analyses, support for six branches varied (Table 2).

A sharp contrast of the high number of putative synapomorphies supporting sister relationships of *Cachrys* clade with Apieae (branch 6, Figure 2) with low “*a la* Bayes” support and posterior probability from CDS analyses is intriguing but will be investigated and discussed elsewhere.

Worth noticing are the short internal branches 1–5, connecting clades of Selineae tribe, *Lefebvrea* clade, Tordyliinae I, *Sinodielsia* clade, and *Coriandrum* + *Nothosmyrnium* + Tordyliinae II clade. The lower support of branches 2 and 4 in the CDS matrix analyses may be attributed to the different amount of phylogenetic information in the CDS and full data matrices, though the different fit of the model to the data or other reasons may also be relevant (discussed in [70]). A level of homoplasy in the CDS matrix is comparable with that in the full matrix (homoplasy index 0.164 and 0.175, respectively). Testing for substitutions saturation in the non-coding and protein-coding sequences showed no presence of saturation (Iss << Iss.c; *p* < 0.0001) in the examined data.

Despite the high support gained in all Bayesian and ML analyses for branch 2, the site concordance factor indicates that actually, there is no overwhelming support for any of the competing topologies. The sCF is close to its lower limit, and this branch is supported by the least number of putative synapomorphies. The sCFs for other short branches are higher but still show a probable presence of conflicting (or a low level of decisive) phylogenetic signal. Nevertheless, this tree is likely the best explanation of relationships of the available plastomes given used models and methods of phylogenetic inference.

As follows from the presented tree, the plastome of *Z. korovinii* has found its place among the plastomes of the *Semenovia* species (Tordyliinae II clade), while *S. graveolens*, *M. komarovii*, and *P. pimpinellifolia* settled down in the Tordyliinae I clade. The plastome of *T. maximum* became a separate lineage closely related to *Heracleum* + *Pastinaca*, and plastomes of two other *Tordylium* species formed a sister group to the *Zosima* + *Semenovia* + *Tetrataenium* clade. Plastomes from *D. suffruticosum* and *N. galbanum*, representing *Lefebvrea* clade of Tordylieae, together formed a sister group to the plastomes from the Selineae tribe. Relationships among the non-Tordylieae samples are identical to the recently presented Apioideae plastome’s phylogeny [26].

Thus, Tordylieae plastomes are of polyphyletic origin and form at least three not closely related lineages, which only partially correspond to the subtribal taxonomy. Plastomes of the Tordyliinae subtribe failed to form a monophyletic group in phylogenomic analyses [20,26,28], and our results are in good agreement with this statement. Plastomes of the genus *Tordylium* also turned out to be not monophyletic on our tree, the type species of the genus *T.* *maximum* separated from the congeneric species in the phylogenetic tree, and their plastomes differ by the presence/absence of insertion in the *trnV*-*rrn16* spacer. It should be noted, that *T. maximum*, a European boreal species, is morphologically remote from the Mediterranean *T. elegans* and *T. pestalozzae* [73]. A possible polyphyly of *Tordylium* had also been suggested earlier based on nrITS data analysis [42,74]. Unfortunately, the genus received little attention in molecular phylogenetic studies and has never been sampled densely, so the questions addressing relationships between *Tordylium* species using nuclear and plastid sequences are still to be answered.

The close affinity of *Symphyoloma*, *Mandenovia*, *Pastinaca*, and *Heracleum* plastomes corresponds well to the results of nrITS studies [42,44,45].

*Ducrosia* and *Kalakia* presented the *Cymbocarpum* clade in our analyses, but despite the presence of insertion in the *trnV*-*rrn16* spacer in both plastomes, the plastome of *K.* *marginata* seems to be not closely related to *D. anethifolia*, which in all analyses is entered in the Tordyliinae II clade (Figure 2, inset), while *K. marginata* was somewhat linked with the Tordyliinae I clade. Support for their placement and the placement itself varied depending on the data set used in the analyses and may change with the addition of other allied plastome sequences, so the plastome of *K. marginata* may represent a lineage in Tordylieae that is separate from Tordyliinae I. We expect that larger sampling with the inclusion of *Cymbocarpum* and other *Tordylium* species will help stabilize the positions of *Ducrosia* and *Kalakia* in the plastome tree.

The *Lefebvrea* clade looks like the only Tordylieae clade keeping monophyly, probably because of the undersampling of the “African peucedanoid group” in our analyses. According to the ITS-taxonomy [31], the group includes ten genera and earlier did not show monophyly in all phylogenetic analyses of the plastid markers (e.g., [75]). A sister relationship of the plastomes from the *Lefebvrea* clade and Selineae tribe, as well as relationships resolved with the short branches 1–5, also require confirmation with the inclusion of yet unsampled allied tribes and clades.

Taking into account the absence of several important apioid superclade lineages (Echinophoreae, *Ormosciadium*, *Opopanax* clade, etc.) and a well-known uncertainty in short branch inference, the plastome relationships in the presented tree should be treated with caution. The most comprehensive study of Apioideae molecular phylogeny using nrITS sequences (2911 accessions) has shown a monophyly of Tordylieae [76]. Recent analysis of nuclear phylogenomic data has confirmed at least monophyly of Tordyliinae and sister relationships of Tordyliinae with the *Lefebvrea* clade [48], which are not recovered in the obtained plastome phylogeny. At the same time, it is difficult to compare results of this investigation with the nuclear phylogenomic study [48] due to differences in taxon sampling: given the unpredictability of plastid phylogeny (i.e., non-monophyly of the *Sinodielsia* clade, Tordyliinae, and Pimpinelleae [26]), the inferred relationships of sampled representatives of the *Lefebvrea* clade should not automatically be applied to the entire *Lefebvrea* clade. Considering plastome and nuclear phylogeny discrepancies in a broader scale, Wen et al. suggested hybridization and incomplete lineage sorting as major sources of observed discordances [26]. Indeed, distant relationships of the Tordyliinae I and Tordyliinae II plastomes hardly can be altered with larger sampling but may be explained with chloroplast capture events, as proposed by Wen et al. [26]. At this stage, we also can hypothesize that ancient chloroplast capture due to hybridization might have played an important role in the *Lefebvrea* clade evolution.

### 2.4. Distribution of Insertion in the trnV-rrn16 Spacer across Tordylieae and Its Allies

The inferred phylogeny showed that all plastomes with *psbA*-*trnH* insertion in the *ycf2*-*trnL* spacer resided within the Tordyliinae I clade, forming a monophyletic group. The distribution of insertions in the *trnV*-*rrn16* spacer, at first sight, assumes at least two appearances and one loss—in the common ancestor of two *Semenovia* species. The mechanisms responsible for the rearrangements at IR junctions are yet not clearly understood; they may involve gene conversion or homologous recombination induced by double-strand break and followed by Holliday junction resolution or illegal recombination [5,77]. In recent years, active studies of the double-strand break repair pathways have provided growing evidence that the error-prone microhomology-mediated mechanisms play important roles in the plastid DNA evolution and may be responsible for structural rearrangements [78,79,80].

Whatever mechanism (or combination of) drives the IR borders in plastome, the absence of the *psbA*-*trnH* insertion in the *trnV*-*rrn16* spacer in *Angelica sinensis* (accessions MT921983 and MT921984), *Semenovia gyirongensis* Q.Y.Xiao & X.J.He (NC_042912), and *Semenovia thomsonii* (C.B.Clarke) Manden. (NC_057441) plastomes is surprising. To check the accuracy of LSC/IRb junction assembly, *S. thomsonii* and *A. sinensis* raw data were retrieved from the sequence read archive (SRR14561442 and SRR13247229, respectively; raw data for *S. gyirongensis* were not accessible). Read mapping showed a drop of coverage depth at the J_LB_ junction, and these two plastomes were reassembled. For *A. sinensis*, three contigs, corresponding to LSC, SSC, and IR regions, were produced, while for *S. thomsonii*, four contigs resulted because of the split of IR. Contigs were joined in a single plastome sequence by virtue of overlapping ends, and the annotation of plastomes revealed the presence of *psbA*-*trnH* insertions in the *trnV*(GAC)-*rrn16* spacer in both plastomes, confirmed with plastome alignment. Thus, in two out of three species, the “disappearance” of the insertion represents a misassembly rather than the real biological phenomenon; it is highly likely that the same is true for *S. gyirongensis*.

To assess the distribution of the insertion across a wider sample of Tordylieae, a short survey of 16 species, including *Ducrosia assadii* Alava, *Cymbocarpum anethoides* DC. ex C.A.Mey., 9 *Semenovia*, 3 *Tordylium,* and 2 *Tetrataenium* species (Supplementary Table S2), was performed using a pair of primers annealed to *trnV*(GAC) and *trnH*(GUG) sequences. The insertion was found in all examined specimens, indicating that its presence is widespread and stable and, surprisingly, that plastomes within *Tordylium* are even more dissimilar than presented in this study. Notably, the *psbA* pseudogene in the insertion in the *Cymbocarpum* and *Kalakia* plastomes are of the same length.

**Figure 3:**
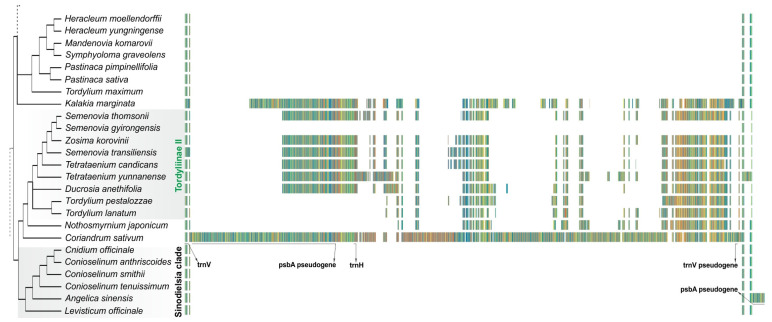
Spacer with an indication of *trnV* pseudogene, *psbA* pseudogene, *trnH* and *trnV* gene sequences, and the distribution of the insertions across the analyzed plastomes. Nucleotide bases in alignment are color-coded; a similar color pattern means a similar sequence. The positions of *Kalakia* and *Ducrosia* are shown as unresolved. Reassembled in this study, the plastomes of *Semenovia thomsonii* and *Angelica sinensis* are presented. Note no matching insertion site in *Angelica*.

Among the insertions in the *trnV*(GAC)-*rrn16* spacer, three are shorter as they do not contain *psbA*-*trnH* sequences (*T. pestalozzae*, *T. lanatum,* and *N. japonicum*, Figure 3).

The alignment of the plastome sequences (Figure 3) shows that the insertion sites in the *trnV*(GAC)-*rrn16* spacer in the clade uniting *Coriandrum* and *Nothosmyrnium* with Tordyliinae II and in the *Sinodielsia* clade in *A.* *sinensis* plastome do not match and occur autonomously. *Coriandrum’s* insertion stands out by its length and differs from *Nothosmyrnium* and Tordyliinae II plastomes by the presence of the *trnV*(GAC) pseudogene in the insertion: this pseudogene is a 5′-truncated copy of the *trnV* gene. It is supposed to emerge as a consequence of a double-strand break repair through the sequence-dependent strand annealing mechanism [55] but might also have arisen earlier by duplication of the *trnV* gene. Considering the fact that the tandem duplication of tRNA (*trnH*(GUG)) genes in the *Piper* plastomes is observed at the IR border [81] (see also Haberle et al. [82]), this is a quite possible scenario. Anyway, as neither *Nothosmyrnium* nor Tordyliinae II plastomes contain the *trnV* pseudogene in IRs, one may conclude that the *psbA*-*trnH* insertion in *Coriandrum* is an independent event. It should also be noted that among all insertions examined, only *Kalakia* showed a high similarity (91%) of the last 70 bases of the non-coding part of the insertion with the *trnK*-*rps16* spacer sequence, suggesting independent J_LA_ shift in the *Kalakia* plastome and its separation from *Ducrosia*.

Thus, within the analyzed plastomes, due to the different positions of the LSC/IRb borders, similar but autonomous J_LA_ shifts have caused three different placements of the *psbA*-*trnH* insertions, and within Tordylieae, the insertions in the *trnV*-*rrn16* spacer happened more than once. At the same time, apparently, it is not so easy for a plastome to be gotten rid of once acquired and captured in IRs insertion without a trace; it should be extremely rare, if ever possible, and these insertions may serve as phylogenetic markers for certain clades. Insertion in the *ycf2*-*trnL*(CAA) spacer within Tordyliinae I seems to have a single origin and inherited by descendants; the origin may be attributed to the *Heracleum* + *Pastinaca* common ancestor on the plastome tree [74]. The mix of “long” (containing *psbA*-*trnH* sequences) and “short” insertions in the *trnV*-*rrn16* spacer at this stage does not allow inferring whether the “long” insertions have shortened or “short” ones have expanded and whether they have a common origin. Unknown positions of the surveyed *Tordylium* species with the “long” insertion and unresolved placement of *D. anethifolia* complicate the situation. Nevertheless, for the members of the *Semenovia* + *Tetrataenium* clade, the insertion also has an obvious single origin, and we believe the same will be found in other members of this clade in the forthcoming plastome studies. During our survey, we indeed determined the presence of the insertion in *S. eriocarpa* (Bornm. & Gauba) Lyskov & Kljuykov; the species was just recently attributed to the genus *Semenovia* (formerly *Seseli elbursense* Pimenov & Kljuykov) based on the morphological features and nrITS and nrETS phylogenetic analysis [83]. The opposite statement cannot be completely true given possible independence of insertion events; however, the presence of the same insertion in the plastome from early branched *Physospermopsis* clade (GenBank accession MW820162, submitted as *Hansenia forbesii*) raises the question of whether the specimen is really not *Tetrataenium* or *Semenovia*.

## 3. Materials and Methods

### 3.1. Plant Material and DNA Extraction

Most samples were collected and identified by M. G. Pimenov and his colleagues during expeditions to the Caucasus, Turkey, and Iran. Additional samples were obtained from E (Royal Botanic Garden, Edinburgh, UK), MW (Moscow State University, Moscow, Russia) herbaria and the carpological collection of the Botanical Garden of Moscow State University. Investigated species and their voucher information are listed in Table 3.

Total genomic DNA (gDNA) from the herbarium material (fruit and leaf) was isolated using either a DNeasy Plant Mini Kit (Qiagen, Germantown, MD, USA) following the manufacturer’s protocol or a modified version of Doyle and Doyle [84].

### 3.2. Genome Sequencing, Plastome Assembly, and psbA-trnH Insertion Survey

The DNA concentration was determined using a Qubit dsDNA HS Assay Kit (Life Technologies, Eugene, OR, USA). Total DNA (1 μg) was fragmented by sonication using a Covaris S220 instrument (Covaris Inc., Woburn, MA, USA). The DNA library was prepared using a TruSeq DNA Sample Prep Kit (Illumina, San Diego, CA, USA) according to the manufacturer’s instructions. The library was sequenced using an Illumina Hiseq2000 or Nextseq500 instrument with a read length of 100 (for Hiseq2000) or 75 bp (for Nextseq500) from each end of the fragment. The resulting reads were processed using the CLC Genomics Workbench software v.5.5. (www.clcbio.com) and Trimmomatic version 0.32 [85]. De novo assembly was performed using a CLC Genomics Workbench and IDBA version 1.1.3 [86]. The resulting contigs, showing homology to plastid genomes, were joined by overlapping ends. To check the accuracy of assembly, trimmed paired reads were mapped to the whole assembled plastome sequence and to the “left” and “right” halves of the circularized plastome using Novoalign v.4.03.00 (www.novocraft.com) followed by manual inspection with Tablet version 1.21.02.08 [87]. This approach allows the definition of IR borders and to confirm the beginning and end of the plastome’s monomer. Genome annotations were performed with the web application GeSeq [88] and checked manually using the Artemis annotation tool [89]; plastome gene maps were drawn using OGDraw version 1.3.1 [90]. Raw data were submitted to GenBank and can be found within the BioProject PRJNA772711.

For the reassembly of *A. sinensis* and *S. thomsonii* plastomes, raw data were retrieved from the sequence read archive (SRR13247229 and SRR14561442, respectively). Data processing, genome assembly, and read mapping were performed as described. Reassembled annotated plastomes of *A. sinensis* and *S. thomsonii* are available in the Third-Party Annotation Section of the DDBJ/ENA/GenBank databases under the accession numbers TPA BK059532 and BK059899, respectively.

In order to confirm the presence of insertions in the *trnV*(GAC)-*rrn16* spacer in assembled plastomes of *Z. korovinii*, *D. anethifolia*, *K. marginata*, *T. lanatum,* and *T. pestalozzae*, we designed primers for PCR amplification and sequencing. To assess the distribution of insertions in the *trnV*(GAC)-*rrn16* spacer, another pair of primers was used (Table 4); their orientation does not allow the amplification when *trnV* and *trnH* are located at the IRa/LSC boundary.

PCR amplification was performed on a Biometra T3000 Thermocycler using an Encyclo PCR kit (Evrogen JSC, Moscow, Russia). Each PCR reaction cycle proceeded as follows: (1) 40 s at 95 °C to denature the double-stranded template DNA; (2) 30 s at 58 °C to anneal primers to single-stranded template DNA; and (3) 30 s at 72 °C to extend primers. The first cycle was preceded by an initial denaturation step of 2 min at 95 °C. To allow completion of unfinished DNA strands and to terminate PCR reaction, a 7-min 72 °C extension period followed the completion of 30 thermal cycles. Each PCR product was purified with a Cleanup Mini kit (Evrogen, Moscow, Russia). PCR products were sequenced (Evrogen, Moscow, Russia) with an ABI 310 Genetic Analyzer (Applied Biosystems, Waltham, MA, USA).

The following 16 species of Tordylieae were surveyed for the presence of insertion in the *trnV*(GAC)-*rrn16* spacer: *Cymbocarpum anethoides* DC.; *Ducrosia assadii* Alava; *Semenovia alaica* Lazkov; *Semenovia dasycarpa* Regel & Schmalh.) Korovin ex Pimenov & V.N.Tikhom.; *Semenovia dichotoma* (Boiss.) Manden.; *Semenovia eriocarpa* (Bornm. & Gauba) Lyskov & Kljuykov; *Semenovia glabrior* (C.B.Clarke) Pimenov & Kljukov; *Semenovia heterodonta* (Korov.) Manden.; *Semenovia pamirica* (Lipsky) Manden.; *Semenovia pimpinellioides* (Nevski) Manden.; *Semenovia tragioides* (Boiss.) Manden.; *Tetrataenium cardiocarpum* (Rech.f. & Riedl) Manden; *Tetrataenium olgae* (Regel & Schmalh.) Manden; *Tordylium apulum* Rchb.; *Tordylium elegans* (Boiss. & Balansa) Alava & Hub.-Mor.; *Tordylium hasselquistiae* DC. Voucher information and GenBank accession numbers are presented in Supplementary Table S2. PCR amplification and sequencing were performed as described.

### 3.3. Phylogenetic Analysis

For phylogenetic analysis, we selected 42 plastid genome sequences from the apioid superclade of the Apioideae subfamily. Earlier, the most representative plastome phylogenetic studies showed that the apioid superclade is a monophyletic group with Careae and Pyramidoptereae tribes branching first [25,26]; therefore, plastomes of *Carum carvi* (Careae), *Crithmum maritimum* L., and *Cyclospermum leptophyllum* (Pyramidoptereae) were used as an outgroup. The Selineae tribe is the most represented tribe in the GenBank plastome database, but as the tribe was also inferred monophyletic [25,26], we restricted ourselves to only four early diverged representatives of Selineae. The list of the retrieved from GenBank plastomes with currently adopted species names and accession numbers are presented in Supplementary Table S3.

Plastome sequences were aligned using MAFFT version 7.471 [91,92] and corrected manually in Bioedit [93]. In aligned plastomes, nineteen small inversions were identified and reverse complemented prior to the analyses. Regions where positional homology could not be firmly determined were excluded along with the gap-rich positions and one copy of the inverted repeat. In addition to the truncated plastome “long”-data matrix, all protein-coding sequences were combined in the “CDS”-data matrix, and both matrices were subjected to phylogenetic analyses.

Plastome’s phylogenetic relations inference was performed using the Bayesian approach and maximum likelihood analysis. The Bayesian inference was performed with the MrBayes version 3.2.6 [94] using four independent runs of 25 million generations and four chains sampling every 1000th generation. The first two million generations were discarded as burn-in, and the remaining trees were combined in a majority-rule consensus tree to obtain the Bayesian posterior probabilities (PP). The maximum likelihood analyses were performed with the IQ-tree version 2.1.1 [95]. Internal branch support was assessed with the approximate likelihood-based approach “*a la* Bayes” [96]. Additionally, to assess variation of phylogenetic signal in our data, the site concordance factor for internal branches—a measure of concordance at the level of individual sites [97]—was estimated in IQ-tree.

To achieve a better fit of the nucleotide substitution models to the data, partitioned analysis was applied to the long data matrix with model parameters unlinked across partitions. The partitions were defined either according to the coding properties—non-coding, protein coding, rRNA, and tRNA genes (four partitions)—or according to the plastome structure—SSC, IR, and LSC regions (three partitions). As sequences in the inverted repeat and single-copy regions have different rates of substitutions [98], the heterotachy model implemented in IQ-tree [99] was used for the three-partition dataset to accommodate a possible substitution rate heterogeneity in the lineages due to IR border shifts. In this case, the region between the *ycf2* and *rrn16* genes was treated as a separate partition; branch support was assessed using 500 nonparametric bootstrap resamplings. Bayesian analysis using partitioned data and covarion model has also been tried, but Markov chains did not show a tendency to converge after 5 million generations, and the analysis was terminated.

The GTR + Γ model of nucleotide substitutions was selected for both data matrices as the most appropriate according to the Akaike information criterion [100] in PAUP version 4.0a [101] and used in unpartitioned and “three partitions” phylogenetic analyses. For analysis of the “four partitions” data, models GTR + F + R3, GTR + F + R2, F81 + F + I, and K2P + I were selected for non-coding, protein coding, rRNA, and tRNA partitions, accordingly, by Bayesian information criterion in the IQ-tree built-in ModelFinder utility [102].

The test for substitutions saturation in the non-coding and protein-coding sequences was performed in DAMBE [103].

## 4. Conclusions

The new data obtained in this study have expanded our knowledge on the range of plastome variability in Apiaceae and demonstrated high mobility of the LSC/IR boundaries within Tordylieae. The more data accumulated, the more chaotic J_LB_ movements within the apioid superclade seem, diminishing the value of the J_LB_ junction type for phylogenetic purposes—closely related plastomes may have different junction types (as in *T. pestalozzae* and *T. lanatum*), and a shared junction type may be confusing when preceding events are hidden (as *A. sinensis*). On the contrary, the presence of the specific rearrangements marks specific lineages and may serve as a phylogenetic marker for certain clades.

With eleven new plastid genomes of Tordylieae, the degree of observed nuclear/plastome discordance has become higher. Plastome clades revealed in our study within Tordylieae coincide more or less with those from nuclear sequences analyses; however, they appeared to be not closely related. Concatenated phylogenetic analyses were unanimous in the highly supported close relationship of the sampled plastomes of the *Lefebvrea* clade with those of the Selineae tribe and in splitting plastomes of the *Cymbocarpum* clade into independent lineages of *Kalakia* and *Ducrosia* with the latter nested within the Tordyliinae II clade. However, despite the high support values obtained, the relationships of plastid genomes revealed in this study should be examined further with larger sampling. Currently, both nuclear transcriptome [104] and plastome phylogenetic analyses reveal short branches separating *Sinodielsia* clade, Tordylieae, and Selineae lineages ([26], this study), and both still suffer from missing lineages and tribes. Possible incomplete lineage sorting and hybridization accompanying fast radiation of these clades may complicate the task of true relationship inference, and additional data and analyses will contribute to our understanding of apioid superclade diversification ways.

## Figures and Tables

**Table 1:** Characteristics of plastid genomes (IR: inverted repeat, LSC: large single-copy region, SSC: small single-copy region) and accession numbers of eleven plastid genomes from Tordylieae tribe species.

Taxon	Size (bp)	LSC Size (bp)	SSC Size (bp)	IR Size (bp)	GC Content (%)	Total Genes	Protein-Coding Genes (Pseudogenes)	tRNA Genes	rRNA Genes	GenBank Accession Number	Mean Coverage Depth (X)
*Dasispermum suffruticosum* (P.J.Bergius) B.L.Burtt	144,902	91,681	16,931	18,145	35.2	126	82 (2)	36	8	OL839263	61
*Ducrosia anethifolia* Boiss.	141,148	98,931	17,523	12,347	37.4	122	79 (2)	35	8	OL839264	25.8
*Kalakia marginata* (Boiss.) Alava	142,155	98,758	17,497	12,950	37.5	122	79 (2)	35	8	OL839265	23.7
*Mandenovia komarovii* (Manden.) Alava	149,345	92,332	17,485	19,764	37.4	127	82 (2)	37	8	OL839266	121
*Notobubon galbanum* (L.) Magee	147,466	93,641	17,443	18,191	37.5	126	82 (2)	36	8	OL839267	51.9
*Pastinaca pimpinellifolia* M.Bieb.	149,758	92,242	17,654	19,931	37.4	127	82 (2)	37	8	NC_027450	82.8
*Symphyoloma graveolens* C.A.Mey.	149,245	92,159	17,516	19,785	37.5	127	82 (2)	37	8	OL839268	115
*Tordylium lanatum* Boiss.	143,402	94,157	17,521	15,862	37.2	124	81 (2)	35	8	OL839269	89.7
*Tordylium maximum* L.	150,103	91,637	17,676	20,395	37.3	126	82 (2)	36	8	OL839270	80.6
*Tordylium pestalozzae* Boiss.	141,830	99,355	17,488	12,493	37.1	122	79 (1)	35	8	OL839271	33.1
*Zosima korovinii* Pimenov	141,644	99,620	17,498	12,263	37.4	122	79 (2)	35	8	OL839272	173

**Table 2:** Support for branches 1–6 gained in phylogenetic analyses of CDS and full data matrices in unpartitioned and partitioned phylogenetic analyses. PP = posterior probability, aBayes = “*a la* Bayes” support, BS = nonparametric bootstrap support, sCF/sDF = site concordance/discordance factor.

Branch	CDS, PP/aBayes	Unpartitioned, PP/aBayes	4 Partitions, PP/aBayes	3 Partitions, PP/aBayes	4 Partitions + Heterotachy, aBayes/BS	sCF/sDF1/sDF2	Putative Synapomorphies
1	1/1	1/1	1/1	1/1	1/97	43.5/28.6/28.9	9
2	0.97/0.95	1/0.99	0.99/	1/1	1/93	36.4/33.2/30.4	2
3	1/1	1/1	1/1	1/1	1/100	42.6/32.8/25.6	20
4	0.99/0.99	1/1	1/1	1/1	1/99	41.4/30.5/28.1	12
5	1/1	1/1	1/1	1/1	1/100	42.6/31.9/25.5	6
6	0.82/0.84	1/1	1/1	1/1	1/99	47.3/16.2/36.5	46

**Table 3:** Voucher information for studied species, including collector name and date. Samples from the carpological collection of the Botanical Garden of Moscow State University are indicated with an asterisk (*).

Species Name	Voucher Number	Locality	Collector, Date
*Dasispermum suffruticosum*	MW0589014	Republic of South Africa, 34°21′ S, 18°55′ E	Pimenov et al., 12 January 2003
*Ducrosia anethifolia*	MW0744172	Iran, prov. Fars, 29°41′ N, 52°45′ E	Pimenov et al., 8 June 2001
*Kalakia marginata*	E №3567	Iran, Iranshakr	Lamond, 1 June 1971
*Mandenovia komarovii*	MW0701533	Russia, Daghestan, left bank of the river Avarskoe Kojsu, near Tlyarata village	Pimenov et al., 15 August 1978
*Notobubon galbanum*	MW0589116	Republic of South Africa, 34°05′ S, 18°25′ E	Pimenov et al., 13 January 2003
*Pastinaca pimpinellifolia*	*	Russia, North Caucasus	Kljuykov et al., 5 August 2005
*Symphyoloma graveolens*	MW0700962	Russia, Daghestan, Andijski distr., Danukh village	Amirhanov, 5 August 1989
*Tordylium lanatum*	*	Turkey, Antalya, Elmali	Pimenov et al., 11 July 2007
*Tordylium maximum*	*	Turkey, prov. Kastamonu, Kure-Inebolu, Ercisler dere	Pimenov et al., 21 August 2008
*Tordylium pestalozzae*	MW0745191	Turkey, Ephesus C1 Izmir: between Ephesus and Mariamane, 37°55′ N, 27°20′ E	Pimenov and Kljuykov, 27 May 1995
*Zosima korovinii*	MW0864736	Kyrgyzstan, bank of river At-Bashi, Baybichetau range, tract Kara-Terek	Pimenov and Kljuykov, 1 August 1987

**Table 4:** Primer sequences used for amplification and sequencing.

Purpose	Primer: Name and Sequence
Insertion in assembled genomes	trnV-rrn16_U: AGTTCGAGCCTGATTATCC
	trnV-rrn16_L: ATTACTTATAGCTTCCTTGTT
Survey of 16 Tordylieae species	trnV-rrn16_U: AGTTCGAGCCTGATTATCC
	trnV-trnH_L: CAATCCACTGCCTTGATCC

## Data Availability

The plastome sequence data presented in this study and the raw data are deposited in GenBank (accession numbers OL839263–OL839272 and NC_027450; BioProject number PRJNA772711; SRA accession numbers SRR16481207, SRR17212745, SRR17212746, SRR17215687–SRR17215693, and SRR17248036).

## Supplementary Materials

The following supporting information can be downloaded at: https://www.mdpi.com/article/10.3390/plants11050709/s1, Figure S1: Alignment of matching sequences of insertions in *ycf2*, *trnV*-*rrn16*, and *ycf2*-*trnL* spacers, Table S1: Gene contents in the eleven Tordylieae plastomes, Table S2: Survey for the presence of insertion in the *trnV*(GAC)-*rrn16* spacer, Table S3: List of chloroplast genome sequences retrieved from GenBank for phylogenetic analysis.
