# Supplementary material for: Plastid Phylogenomic Analysis of Tordylieae Tribe (Apiaceae, Apioideae)
Source: Plants (Basel). 2022 Mar 7;11(5):709. doi: 10.3390/plants11050709 (PMC8912408; doi:10.3390/plants11050709)
Supplement: Supplementary file 1 [file plants-11-00709-s001.zip › Suppl_Table S1.List of genes.pdf]

**Table S1**. Gene contents in the eleven Tordylieae plastomes.

| **Category** | **Gene Groups** | **Gene Names** |
|---|---|---|
| Self-replication | Ribosomal RNA genes (rRNA) | *rrn5* [a], *rrn4.5* [a] , *rrn16* [a], *rrn23* [a] |
| | tRNA genes | *trnA-UGC* [a] , *trnC-GCA*, *trnD-GUC*, *trnE-UUC* [a], *trnF-GAA*, *trnG-GCC*, *trnG-UCC* [a], *trnH-GUG* [a1, a2], *trnI-CAU*, *trnI-GAU* [a], *trnK-UUU*, *trnL-CAA* [a1, a3], *trnL-UAA*, *trnL-UAG* , *trnfM-CAU*, *trnM-CAU*, *trnN-GUU* [a], *trnP-UGG*, *trnQ-UUG*, *trnR-ACG* [a], *trnR-UCU*, *trnS-GCU*, *trnS-GGA*, *trnS-UGA*, *trnT-GGU*, *trnT-UGU*, *trnV-GAC* [a1, a3, a4, a5], *trnV-UAC* [a] , *trnW-CCA*, *trnY-GUA* |
| | Ribosomal small subunit | *rps2*, *rps3*, *rps4*, *rps7* [a1, a3, a4], *rps8*, *rps11*, *rps12_5'end*, *rps12_3'end*, *rps14*, *rps15*, *rps16*, *rps18*, *rps19* |
| | Ribosomal large subunit | *rpl2*, *rpl14*, *rpl16*, *rpl20*, *rpl22*, *rpl23*, *rpl32*, *rpl33*, *rpl36* |
| | DNA-dependent RNA polymerase | *rpoA*, *rpoB*, *rpoC1*, *rpoC2* |
| Photosynthesis | Large subunit of rubisco | *rbcL* |
| | Photosystem I | *psaA*, *psaB*, *psaC*, *psaI*, *psaJ*, *paf1*,*paf2* |
| | Photosystem II | *psbA*, *psbB*, *psbC*, *psbD*, *psbE*, *psbF*, *psbH*, *psbI*, *psbJ*, *psbK*, *psbL*, *psbM*, *psbN*, *psbT*, *psbZ* |
| | NADH dehydrogenase | *ndhA*, *ndhB* [a1, a3], *ndhC*, *ndhD*, *ndhE*, *ndhF*, *ndhG*, *ndhH*, *ndhI*, *ndhJ*, *ndhK* |
| | Cytochrome b/f complex | *petA*, *petB*, *petD*, *petG*, *petL*, *petN* |
| | ATP synthase | *atpA*, *atpB*, *atpE*, *atpF*, *atpH*, *atpI* |
| Other | Maturase | *matK* |
| | Subunit of acetyl-CoA carboxylase | *accD* |
| | Envelope membrane protein | *cemA* |
| | ATP dependent protease subunit P | *clpP* |
| | Translational initiation factor | *infA* |
| | c-type cytochrome synthesis | *ccsA* |
| | Conserved open reading frames (*ycf*) | *ycf1*, *ycf2* |

[a] genes in the IR regions (two copies in the plastome)
[a1] duplicated gene in the IR regions of *Pastinaca*, *Mandenovia*, *Symphyoloma*
[a2] duplicated gene in the IR regions of *Kalakia*, *Ducrosia*, *Zosima*
[a3] duplicated gene in the IR regions of *Dasispermum*, *Notobubon*, *Tordylim maximum*
[a4] duplicated gene in the IR regions of *Tordylium lanatum*
[a5] duplicated gene in the IR regions of *Tordylim pestalozzae*
