# Supplementary material for: Plastid Phylogenomic Analysis of Tordylieae Tribe (Apiaceae, Apioideae)
Source: Plants (Basel). 2022 Mar 7;11(5):709. doi: 10.3390/plants11050709 (PMC8912408; doi:10.3390/plants11050709)
Supplement: Supplementary file 1 [file plants-11-00709-s001.zip › Suppl_Table S2.Survey.pdf]

Table S2. Survey for the presence of insertion in trnV(GAC)-rrn16 spacer.

Species name	Genbank accession	Length	Voucher	Country	Collection date	Collector
Cymbocarpum anethoides DC.	OL792759	832	MW0700433	Georgia	16-Jun-1988	M.V. Kostyleva
Ducrosia assadii Alava	OL675434	448 bp	E950	Iran	27-Apr-1977	R. Alava
Semenovia alaica Lazkov	OL631192	546 bp	MW0594097	Kyrgyzstan	15-Jun-2006	G. A. Lazkov
Semenovia dasycarpa Regel & Schmalh.) Korovin ex Pimenov & V.N.Tikhom.	OL675428	547 bp	MW0897150	Tajikistan	07-Aug-2012	E. V. Kljuykov & U. A. Ukrainskaya
Semenovia dichotoma (Boiss.) Manden.	OL675432	533 bp	MW236	Iran	07-Jun-2001	M. G. Pimenov, E. V. Kljuykov, A. K. Sytin, F. Ghahremani-Nejad
Semenovia eriocarpa (Bornm. &Gauba) Lyskov & Kljuykov	OL792760	547	MW0757072	Iran	26-Jun-2019	D. Lyskov
Semenovia glabrior (C.B.Clarke) Pimenov & Kljukov	OL631190	544 bp	MW s.n.	North India	26-Jul-2017	U. A. Ukrainskaya
Semenovia heterodonta (Korov.) Manden.	OL675429	547 bp	MW0897143	Tajikistan	31-Jul-2012	E. V. Kljuykov & U. A. Ukrainskaya
Semenovia pamirica (Lipsky) Manden.	OL675430	535 bp	LE27	Tajikistan	21-Jul-1956	S. Ikonnikov
Semenovia pimpinellioides (Nevski) Manden.	OL675427	551 bp	MW0864993	Uzbekistan	14-Aug-2010	M. G. Pimenov & E. V. Kljuykov
Semenovia tragioides (Boiss.) Manden.	OL631191	547 bp	MW0754182	Iran	15-Jun-2014	E. V. Kljuykov & D. F. Lyskov
Tetrataenium cardiocarpum (Rech.f. & Riedl) Manden.	OL675433	543 bp	MW1259	Pakistan	22-Aug-1990	U. Schishkoff
Tetrataenium olgae (Regel & Schmalh.) Manden	OL675431	550 bp	MW0745065	Afghanistan	10-Jul-1974	I. Gubanov
Tordylium apulum Rchb.	OL675435	529 bp	MW0745183	Turkey	22-May-1995	M. G. Pimenov & E. V. Kljuykov
Tordylium elegans (Boiss. & Balansa) Alava & Hub.-Mor.	OL675436	538 bp	MHA6694	Turkey	19-May-1967	R. Alava
Tordylium hasselquistiae DC.	OL675437	535 bp	MHA6605	Turkey	12-May-1967	R. Alava
