# Supplementary material for: Plastid Phylogenomic Analysis of Tordylieae Tribe (Apiaceae, Apioideae)
Source: Plants (Basel). 2022 Mar 7;11(5):709. doi: 10.3390/plants11050709 (PMC8912408; doi:10.3390/plants11050709)
Supplement: Supplementary file 1 [file plants-11-00709-s001.zip › Suppl_Table S3.List of genomes.pdf]

Table S3. List of chloroplast genome sequences retrieved from Genbank for phylogenetic analysis.

Species name	Acc. number	Species name
Anethum graveolens	NC_029470	
Angelica sinensis	reassembly	
Apium graveolens	MK036045	
Carum carvi	NC_029889	
Cnidium officinale	NC_039760	
Conioselinum anthriscoides (H.Boissieu) Pimenov & Kljuykov, (Skvortsovia 2(1): 33 (2015).	NC_038088	= *Ligusticum sinense*
Conioselinum pteridophyllum (Franch.) Lavrova, (Abstr. Int. Sci. Conf. Syst. Higher Pl. 66 (2002)	NC_049056	= *Ligusticum pteridophyllum*
Conioselinum smithii (H.Wolff) Pimenov & Kljuykov, (Willdenowia 33(2): 369 (2003)	MT561037	= *Ligusticum jeholense*
Conioselinum tenuissimum (Nakai) Pimenov & Kljuykov, (Willdenowia 33(2): 373 (2003)	NC_029394	= *Ligusticum tenuissimum*
Coriandrum sativum	NC_029850	
Crithmum maritimum	NC_015804	
Cyclospermum leptophyllum	MT561040	
Foeniculum vulgare	KR011054	
*Heracleum moellendorffi*i	NC_042242	
Heracleum yungningense	NC_047287	
Levisticum officinale	MT561024	
Ligusticopsis hispida (Franch.) Lavrova & Kljuykov, (Bot. Zhurn. (Moscow & Leningrad) 79(10): 106 (1994).	NC_049053	= *Ligusticum hispidum*
Ligusticopsis involucrata (Franch.) Lavrova, (Turczaninowia 20(2): 171 (2017)	NC_049054	= *Ligusticum involucratum*
Nothosmyrnium japonicum	MT561036	
Pastinaca sativa	MT561034	
Petroselinum crispum	NC_015821	
Prangos fedtschenkoi	KY652265	
Prangos lipskyi	KY652266	
Prangos trifida	MG386251	
Semenovia gyirongensis	NC_042912	
Semenovia thomsonii	reassembly	
Semenovia transiliensis	NC_045182	
Seseli montanum	NC_027451	
Seseli mucronatum (Schrenk) Pimenov & Sdobnina, (Byull. Moskovsk. Obshch. Isp. Prir., Otd. Biol. 78(4): 139 (1973).	NC_049058	= *Ligusticum thomsonii*
Tetrataenium candicans	MK522402	
Tetrataenium yunnanense	NC_045183	
