# Supplementary figures and images for: Plastid Phylogenomic Analysis of Tordylieae Tribe (Apiaceae, Apioideae)
Source: Plants (Basel). 2022 Mar 7;11(5):709. doi: 10.3390/plants11050709 (PMC8912408; doi:10.3390/plants11050709)

Figure S1. Alignment of matching sequences of insertions in ycf2, trnV-rrn16 and ycf2-trnL spacers.

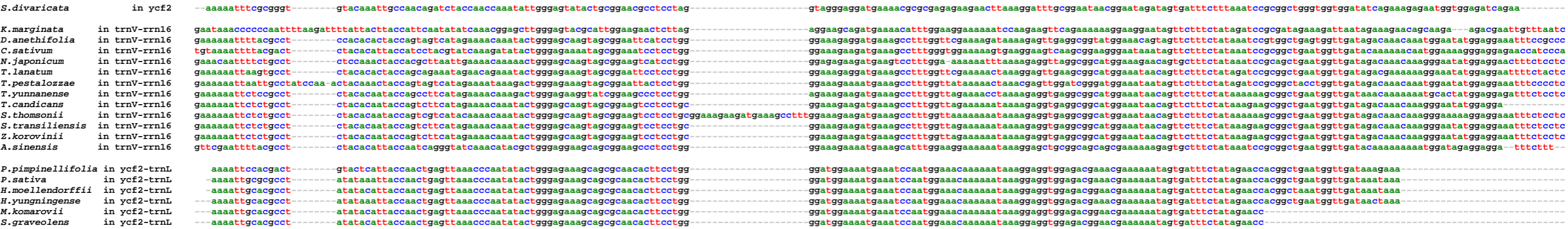

Supplement: Supplementary file 1 [file plants-11-00709-s001.zip › Suppl_Figure S1.align.pdf]
